# Investigation of materials for the development of new molecular and cellular antiviral and antimalignancy vaccines and immunization methods: a pilot study

**DOI:** 10.5114/bta/210359

**Published:** 2025-12-06

**Authors:** Iskra Sainova, Vera Kolyovska, Radka Hadjiolova, Andrey Petrov, Dimitrina Dimitrova-Dikanarova, Tzvetanka Markova

**Affiliations:** 1 Institute of Experimental Morphology, Pathology and Anthropology with Museum to Bulgarian Academy of Sciences, Sofia, Bulgaria; 2 Department of Pathophysiology, Medical University of Sofia, Bulgaria; 3 Department of Toxicology, University Hospital “Joanna Queen”, Sofia, Bulgaria; 4 Department of Biology, Medical University of Sofia, Bulgaria; 5 Department of Pharmacology and Toxicology, Medical University of Sofia, Bulgaria

**Keywords:** DNA-viruses and RNA-viruses, intracellular and extracellular viral forms, fusion processes activation, immune cells progenitors

## Abstract

**Background:**

Changes in biomolecules under the influence of chemical and physical factors on cells, tissues, and whole organisms are investigated.

**Materials and methods:**

*In vitro*-incubated bovine embryonic cells were inoculated with low titers (high dilutions of viral suspensions) of vaccine avipoxviral strains. Mouse embryonic fibroblasts were co-cultivated with mouse malignant myeloma cells (P3-X63-Ag8) transfected by recombinant DNA plasmid or preincubated in culture fluid from prior incubation of the same cells. Sub-populations of virus-inoculated, co-cultivated, and preincubated cell cultures were frozen in the presence of the cryoprotectant dimethylsulfoxide (DMSO), subsequently thawed, and re-incubated. Newly formed cell monolayers were inoculated with extracellular and intracellular forms of each viral strain, both before and after exposure to DMSO and drastic temperature changes. Extracellular forms were derived from the cultural fluids of inoculated cell cultures, while intracellular forms were obtained from suspensions of mechanically scraped virus-inoculated cells.

**Results:**

Exchange of nucleotide (DNA and/or RNA) fragments between cellular and viral genomes, as well as between genomes of separate cells, was suggested. These changes were explained by activated fusion induced by the organic detergent (DMSO) combined with drastic temperature changes. Such processes could provide vectors for gene-engineering manipulations and the development of molecular (DNA-based, RNA-based, and/or protein-based) antiviral and antimalignant vaccines. Production of immune molecules by nonimmune cell types under appropriate conditions, such as the presence of immunomodulators, was also proposed.

**Conclusions:**

The results suggest the possibility of nucleotide (DNA and/or RNA) fragment exchange between separate cells, as well as between cells and virions. Nonimmune cells demonstrated the capacity to produce immune molecules under appropriate conditions.

## Introduction

The property of viruses to acquire foreign DNA and/or RNA material has positioned them as appropriate candidates for use as vectors in the design of novel molecular (DNA-based, RNA-based, and/or protein-based) anti-malignancy and anti-infectious vaccines, as well as in recombinant DNA constructs for gene-engineering manipulations through the transfer of appropriate DNA and/or RNA fragments, including into different cells (Chen et al. 2003; Palese and Roizman 1996; Xiong et al. 2005). Maximally safe applications should be ensured by eliminating genes responsible for infections or malignant transformations (Gonçalves 2005; McLaughlin et al. 1988; Wong et al. 2013), as well as by applying low initial infectious titers (i.e., high dilutions of viral suspensions) of heterologous attenuated vaccine strains relative to the respective species (Sainova et al. 2005, 2023). Reports of similar applications involving bacterial plasmids (Domi and Moss 2005) or yeast genomes have also been published (Brachmann et al. 1998).

The designed DNA viral vectors should contain specific restriction sites, and the respective DNA fragment(s) of interest should be obtained through treatment with restriction enzymes (particularly bacterial endonucleases). Possible modifications may enhance the expression level of the inserted foreign fragment(s), either in the promoter or at the insertion site (the locus where the copy of the respective gene of interest is integrated) (Galindo et al. 2001).

RNA viruses have also been proposed for many gene therapy applications and vaccine development strategies (Barrette et al. 2000; Lundstrom 2019). Technologies based on viruses with RNA genomes have been successfully applied for reprogramming, *trans*-differentiation, gene editing, and gene therapy of various diseases, including neoplasms, as well as for the production of anti-infectious and antimalignancy vaccines (Schott et al. 2016). In the application of retroviral particles, the respective genes of interest have been shown to incorporate directly into the viral RNA genome or as nonviral RNA, while nonsegmented negative-strand RNA viruses such as alphavirus and flavivirus-derived vectors have demonstrated prolonged expression through replication of viral RNA encoding the genes of interest. The precision of RNA interference (RNAi) in targeting and degrading unwanted cellular and/or viral RNAs has also led to the development of siRNA-based treatments for various diseases (Kang et al. 2023).

Production of inflammatory mediators not only by infiltrating immune cells but also by nonimmune cells in response to specific stimuli has been established (Sisto and Lisi 2023). The possibility that non-immune cells may possess immunological memory has also been suggested (Hamada et al. 2019). In this context, certain nonimmune cells have been shown to exhibit characteristics of trained immunity (Tercan et al. 2021). Various epigenetic and metabolic mechanisms have been proposed to underlie the ability of cells – particularly in the early phases of maturation and differentiation – to acquire trained immunity, depending on the respective cell type (Acevedo et al. 2021). Myeloid and lymphoid cells have also demonstrated the capability to differentiate in appropriate directions for incorporating coding nucleotide fragments of antiviral or antimalignancy antigens (Cheng et al. 1998). Furthermore, some myeloid cells (particularly neutrophils) have shown the ability to release their own DNA as extracellular traps to capture and eliminate pathogens (Neeli et al. 2009; Neubert et al. 2018). The presence of single cells with marginated chromatin and/or large multinucleated cells has been characterized as an important diagnostic criterion for infection, inflammation, and/or malignancy (Segura et al. 2018).

Considering the diverse influences of chemical and physical factors on cells, tissues, organs, and organisms, changes in many properties of biomolecules have been demonstrated. For example, various organic detergents and drastic temperature shifts have been shown to alter the properties of cellular membrane structures and activate fusion processes (de Ménorvan et al. 2012; Frey et al. 1995; Jacob 1986; Norwood et al. 1976).

In this context, the main goal of the current study was to develop *in vitro* methods for the incubation of viral strains with DNA and RNA genomes in laboratory-cultivated mammalian cells. Accordingly, the central idea of the study was directed toward the development of techniques for deriving immune cells and viral vectors for gene-engineering manipulations, including the design of new antimalignancy and antiviral molecular (DNA-based, RNA-based, and/or protein-based) vaccines.

## Materials and methods

### Cellular in vitro cultures

Embryonic cells from the mammalian lines EBTr and 3T3, derived from embryonic bovine trachea cells and mouse embryonic fibroblasts, respectively, were used. Cells of both types were incubated at an initial volume of 3 × 10^4^/1 ml of culture fluid. The growth medium was a 1 : 1 combination of ParkerE-199 (Sigma) and Iscove’s modification of Dulbecco’s medium (IMDM, Sigma), supplemented with 25 mM HEPES buffer (Sigma), 5% fetal bovine serum (FBS, Sigma), and an antibiotic mixture (100 IU/ml penicillin, Sigma, and 100 μg/ml streptomycin, Sigma). At the same time, *P3-X63-Ag8* mouse malignant myeloma cells transfected with a recombinant DNA plasmid were cultured *in vitro* under similar conditions. These cells were incubated in RPMI 1640 (Sigma) supplemented with 5% FBS (Sigma), 100 IU/ml penicillin (Sigma), and 100 μg/ml streptomycin (Sigma). All cell cultures were maintained in a humidified 5% CO_2_/95% air incubator at 37°C. Separate subpopulations of the virus-inoculated and preincubated embryonic cells of both types were frozen in the presence of the cryoprotectant dimethylsulfoxide (DMSO), subsequently thawed, and re-incubated.

### In vitro incubation of viral strains

Separate subpopulations of *in vitro*-incubated EBTr mammalian cells were inoculated (24-well Nunclon plates; Space Saver Flow Lab.; Linbro) as semi-confluent monolayers with low initial infectious titers (i.e., high dilutions of viral suspensions) of the DNA vaccine avian poxvirus strains FK (fowl) and Dessau (pigeon) (family *Poxviridae*). After absorption for 45 min at room temperature, the inoculated cell cultures were washed three times with 1 ml per well of phosphate-buffered solution (PBS, pH 7.2), after which 1 ml/well of supporting medium was added.

The intracellular forms of both vaccine strains were obtained by mechanically scraping the monolayers of virus-inoculated cells, while the extracellular forms were derived from the culture fluids of the same inoculated cells. After isolating the two forms of each viral strain, newly formed monolayers of embryonic bovine cells in 24-well plates were inoculated with each form. Part of the seeded cell monolayers were inoculated with the two forms of each viral strain before exposure to the organic detergent DMSO plus drastic temperature changes, and the other part after such treatment.

Similarly, subpopulations of *in vitro*-incubated 3T3 mouse embryonic cells were preincubated in culture fluid previously used for incubation of *P3-X63-Ag8* mouse malignant myeloma cells transfected with recombinant DNA plasmid (after prior centrifugation and filtration). These myeloma cells contain retroviral genome fragments (*Retroviridae* family) (Kearney et al. 1979). Another subpopulation of 3T3 fibroblasts was co-cultivated with the same malignant cells (by addition of both culture fluid and cell suspension). Again, separate subpopulations of the mixed cultures, as well as the 3T3 cells pre-incubated in the culture fluid of mouse malignant myeloma cells, were frozen in the presence of DMSO, subsequently thawed, and re-incubated.

### Statistical assay

The degree of the assessed cytopathogenic effect (CPE) was compared across the separate virus-inoculated cell cultures and at different times post-inoculation. The CPE was evaluated for both intracellular and extracellular forms of each of the two vaccine viral strains, before and after exposure to DMSO and drastic temperature changes. Data were subsequently processed using statistical software. Values were expressed as mean ± standard deviation (SD), and Student’s *t*-test was applied. Differences were considered statistically significant at *p* < 0.01 and *p* < 0.05.

## Results

### Inoculation of in vitro-incubated cells with viruses possessing a DNA genome, viral replication and suggestion of provirus presence in the cellular genome

**Figure 1:**
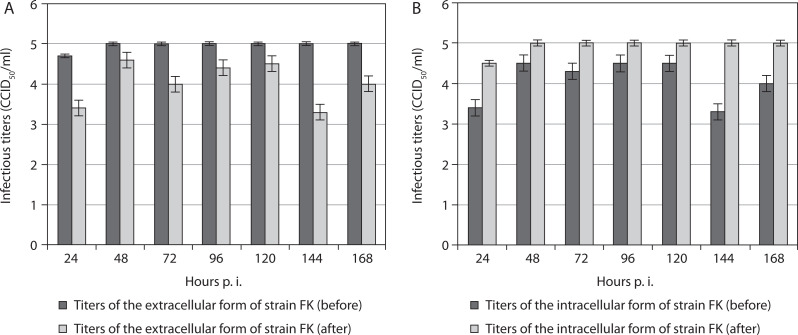
Titers of extracellular (**A**) and intracellular (**B**) forms of the vaccine fowlpox viral strain FK incubated in mammalian cells, before and after freezing in the presence of the cryoprotectant DMSO, followed by thawing and re-incubation. Data were obtained by comparing values with noninoculated controls and processed using software analysis (± SD, *p* < 0.01)

In all cases, the titers of the extracellular form of the fowl avian poxvirus strain FK before freezing in the presence of the organic detergent DMSO, followed by thawing and re-incubation, were significantly higher than after the procedure (Figure 1A). This tendency was observed from the 24^th^ to the 168^th^ hour postinoculation (p.i.). These results suggest decreased titers of the extracellular form of this vaccine strain after the procedure. Overall, the titer of the extracellular form of strain FK increased after the 24^th^ hour p.i. and remained constant from the 48^th^ to the 168^th^ hour p.i., unlike the observed variations in titers at the same time points before manipulation. In contrast, the intracellular form of strain FK demonstrated increased titers after the manipulation compared with before (Figure 1B). Similarly, significantly increased titers of the intracellular form were observed after the 24^th^ hour p.i., remaining constant from the 48^th^ to the 168^th^ hour p.i.

**Figure 2:**
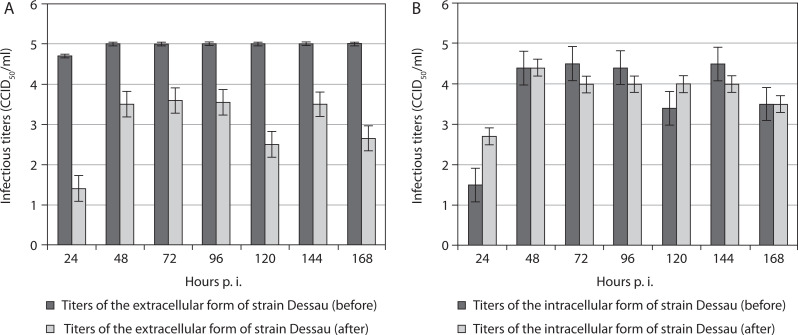
Titers of extracellular (**A**) and intracellular (**B**) forms of the vaccine pigeonpox viral strain Dessau incubated in mammalian cells, before and after freezing in the presence of the cryoprotectant DMSO, followed by thawing and re-incubation. Data were obtained by comparing values with noninoculated controls and processed using software analysis (± SD, *p* < 0.01)

For the vaccine pigeon poxvirus strain Dessau, titers of the extracellular form were significantly higher before freezing in the presence of DMSO and subsequent thawing than after it, across all time points from the 24^th^ to the 168^th^ hour p.i. (Figure 2A). Again, after the 24^th^ hour p.i., a decrease in the extracellular titer was noted, which remained constant from the 48^th^ to the 168^th^ hour p.i. This trend was not observed in the intracellular form of the Dessau strain. Instead, despite a reduction after exposure to DMSO and drastic temperature changes, its intracellular titers increased from the 24^th^ to the 168^th^ hour p.i. compared with corresponding values before the manipulation (Figure 2B). The extracellular form of strain Dessau showed higher titers than its intracellular form only at the 120^th^ hour p.i. At the 48^th^ and 168^th^ hours, equal titers of the intracellular form were observed before and after manipulation (Figure 2B). At the 24^th^, 72^nd^, 96^th^, and 144^th^ hours p.i., the intracellular titers of the same strain were higher before the manipulation than after it.

### Inoculation of in vitro-incubated cells with viruses with RNA (retroviral) genome, viral replication and suggestion of provirus presence in the cellular genome

**Figure 3:**
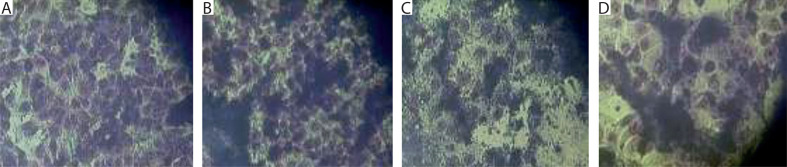
*In vitro* cultures of mouse embryonic 3T3 fibroblasts were subjected to different conditions. **A**) Control culture. **B**) Initially, myeloid-like and lymphoid-like progenitors derived from stem-like cells in the 3T3 line after preincubation in culture fluid from *P3-X63-Ag8* mouse malignant myeloma cells transfected with recombinant DNA plasmid. **C**) Initial myeloid-like and lymphoid-like progenitors, as well as later phagocyte-like and plasmacyte-like cells, derived from stem-like cells in the 3T3 line after co-cultivation with *P3-X63-Ag8* transfected myeloma cells (culture fluid plus cell suspension). **D**) Hybrid cells derived from 3T3 fibroblasts pre-incubated in culture fluid from *P3-X63-Ag8* transfected myeloma cells, frozen in the presence of DMSO, thawed, and re-incubated. Actively proliferating myeloid-like and lymphoid-like progenitors with oval shape and increased size are visible. Fixed light-microscopy preparations, stained with H/E; magnification: 150×

Upon preincubation of mouse embryonic fibroblasts from the 3T3 line in culture fluid from *P3-X63-Ag8* mouse malignant myeloma cells transfected with recombinant DNA plasmid (after centrifugation and filtration), the appearance of initial myeloid-like and lymphoid-like cells was observed (Figure 3B). These features were absent in the control culture of 3T3 fibroblasts (Figure 3A). Similar changes were also noted during co-cultivation of 3T3 cells with mouse malignant myeloma cells (culture fluids plus cell suspensions) containing retroviral genome material (Figure 3C). In this case, signs of further differentiation toward phagocyte-like and plasmatic cell-like morphology were established.

The described changes were mainly expressed as an increase in the size of the nonmalignant cells, the acquisition of an oval shape, and, in some cases, the formation of membrane/cytoplasmic pseudopodia (Figure 3B, C). After freezing the mixed cultures in the presence of DMSO, followed by thawing and re-incubation, cells with increased size and a rounded shape were observed (Figure 3D). These changes could be explained by a possible exchange of nucleotide (DNA and/or RNA) fragments between the cellular and retroviral genomes.

## Discussion

Technologies based on viruses with both DNA and RNA genomes have been successfully applied in reprogramming, trans-differentiation, gene editing, anti-infectious and antimalignancy vaccination, and gene therapy for various diseases, including neoplasms (Acevedo et al. 2021; Barrette et al. 2000; Chen et al. 2003; Cheng et al. 1998; Galindo et al. 2001; Gonçalves 2005; Guenechea et al. 2000; Hamada et al. 2019; Kang et al. 2023; McLaughlin et al. 1988; Palese and Roizman 1996; Schott et al. 2016; Sisto and Lisi 2023; Tercan et al. 2021; Wong et al. 2013; Xiong et al. 2005; Zhang et al. 2002; Zhang et al. 2004). In all cases, the necessity of modifying viruses to eliminate their ability to cause disease has been demonstrated (Johnson et al. 2022). To achieve this, viral genome fragments responsible for viral pathology should be removed (Gonçalves 2005). At the same time, the influence of cellular genes has also been established (Wong et al. 2013). Many conserved genes have been identified between cattle and mouse (Li et al. 2012), as well as between mouse and human (Breschi et al. 2017).

The observed signs of initial myeloid-like and lymphoid-like differentiation could be explained by the presence of a subpopulation of stem-like cells within the cell line that retain the ability to differentiate in various directions (Kobari et al. 2000; Kyba et al. 2003). The capacity of low-differentiated stem/progenitor cells has been identified as an important property for resetting both innate and adaptive immunity from an inflammatory state to a repair state once the virus has been cleared (Maguire 2021). Reports have also indicated that induced pluripotent stem cells can differentiate into functional immune effector cells in the presence of appropriate growth factors and cytokines (Kao et al. 2023).

Other studies have demonstrated the protective potential of human gene therapy against malignancies and other diseases through gene transfer using mesenchymal stem cells from bone marrow transfected with retroviral/lentiviral constructs (Guenechea et al. 2000; Zhang et al. 2002; Zhang et al. 2004). Variations in the expression and function of innate immune sensors and antiviral effectors across species, cell types, differentiation phases, and environmental conditions have also been described (Goldstein and Scull 2022).

In addition to the established effects of fibroblasts as key factors in the tumor microenvironment, neuronal cells and their components, soluble cytokines, exosomes, and the microbiome have also been identified as important influences on tumor immunotherapy (Li et al. 2021). The role of long noncoding RNAs (lncRNAs) in lineage commitment of immune cells and in shaping immune responses has also been proposed (Ahmad et al. 2020). Furthermore, the concept of “intrinsic antiviral immunity”, referring to the cell’s internal protective response to infection, has been advanced (Murray et al. 2018; Yan and Chen 2012).

Although trained immunity is beneficial against infections and malignancies, evidence also indicates that its inappropriate induction by certain endogenous stimuli can lead to aberrant inflammation (Ochando et al. 2023). In this way, an increased risk of inappropriate inflammation, allergic and/or atopic reactions, autoimmune diseases, immunodeficiency disorders, and hypersensitivity reactions may be proposed (Marshall et al. 2018).

## Conclusions

According to the results of this pilot study, initial steps were taken toward the development of new molecular (DNA-based, RNA-based, and/or protein-based antimalignancy and anti-infectious vaccines, as well as vectors for gene-engineering manipulations. The data obtained were based on the demonstrated activation of fusion processes – between cells of the same type or different types, as well as between cells and viral particles – under the influence of organic detergents combined with drastic temperature changes. In this way, the exchange of nucleotide (DNA and/or RNA) fragments between viruses and cells in both directions was proposed. Additionally, signs of initial differentiation of low-differentiated embryonic cells into myeloid-like and lymphoid-like lineages under the influence of malignant cells or antigens, infectious agents and antigens, and immunomodulators were suggested. Future investigations are required.
